# The Fitness Cost of Fluoride Resistance for Different *Streptococcus mutans* Strains in Biofilms

**DOI:** 10.3389/fmicb.2017.01630

**Published:** 2017-08-28

**Authors:** Yanling Cai, Ying Liao, Bernd W. Brandt, Xi Wei, Hongyan Liu, Wim Crielaard, Cor Van Loveren, Dong Mei Deng

**Affiliations:** ^1^Department of Operative Dentistry and Endodontics, Guanghua School of Stomatology, Hospital of Stomatology, Sun Yat-sen University Guangzhou, China; ^2^Guangdong Province Key Laboratory of Stomatology Guangzhou, China; ^3^Department of Preventive Dentistry, Academic Centre for Dentistry Amsterdam, University of Amsterdam and Vrije Universiteit Amsterdam Amsterdam, Netherlands; ^4^State Key Laboratory of Oral Diseases, West China Hospital of Stomatology, Sichuan University Chengdu, China

**Keywords:** *Streptococcus mutans*, fluoride resistance, chlorhexidine, low pH, sodium fluoride, fitness cost, biofilm

## Abstract

The cariogenic bacterium *Streptococcus mutans* can develop stable resistance to fluoride through chromosomal mutations *in vitro*. Fluoride-resistant *S. mutans* has seldom been isolated in clinical settings, despite the wide application of fluoride in oral-care products. One explanation is that the fluoride-resistant *S. mutans* strains have decreased fitness. However, so far, there has been no conclusive evidence to support this idea. The aim of this study was to investigate the fitness cost of 48-h biofilms of two fluoride-resistant *S. mutans* strains, UF35 and UA159-FR (UAFR), using the wild-type fluoride-sensitive strain UA159 as a reference. The engineered UF35 strain contains one point mutation, whereas UAFR, selected from NaF-containing agar plates, has multiple chromosomal mutations. All biofilms were formed for 48 h under a constantly neutral pH or a pH-cycling (8 h of neutral pH and 16 h of pH 5.5) condition in the absence of fluoride. The biomass of the biofilms was quantified with a crystal violet assay. The biofilms were also treated with chlorhexidine or solutions at pH 3.0, after which their lactic acid production was quantified. Compared to the UF35 and UA159 biofilms, the biomass of UAFR biofilms was two–four fold higher, and the UAFR biofilms were more resistant to chlorhexidine and low pH in terms of lactic acid production. No difference in biomass and lactic acid production was detected between UF35 and UA159 biofilms. The fluoride resistance of UAFR and UF35 strains in biofilms was further confirmed by treating the biofilms with NaF solutions. The level of NaF resistance of the three biofilms is generally ranked as follows: UAFR > UF35 > UA159. In conclusion, there is indeed a fitness consequence in UAFR, but surprisingly, this fluoride-resistant strain performs better than UF35 and UA159 under the described conditions. In addition, UF35 did not display a reduced fitness; it performed as well as the wild-type fluoride-sensitive strain.

## Introduction

Fluoride is the most effective caries-preventive agent. Since the 1940s, it has been added to water supplies and to daily oral care products, such as toothpaste, mouthwash, and dental floss (Birkeland and Torell, 1978). It not only protects dental hard tissues by inhibiting demineralization and enhancing remineralization but also functions as an antimicrobial agent (Van Loveren, 2001; Ten Cate, 2004). Fluoride is able to suppress bacterial growth and metabolism (Brown et al., 1980; Van Loveren et al., 1991b). To counteract this suppression, several oral bacterial species, including *Streptococcus mutans*, have acquired resistance to fluoride in the presence of a high fluoride concentration (Hamilton, 1969; Streckfuss et al., 1980; Bunick and Kashket, 1981; Sheng and Liu, 2000).

*Streptococcus mutans* has been recognized as one of the major cariogenic microorganisms because it can produce a large amount of lactic acid when given sugar; it is able to tolerate a low pH and it strongly forms biofilms in the presence of sucrose (Loesche, 1986). In a laboratory setting, several fluoride-resistant *S. mutans* strains have been isolated under high fluoride concentration conditions. These strains were able to grow at fluoride concentrations at least three times higher than the concentration optimal for fluoride-sensitive *S. mutans* strains. This acquired fluoride resistance is rather stable as it persisted for at least 50 generations when cultivated without fluoride. We previously identified multiple single nucleotide mutations in the genome of a fluoride-resistant *S. mutans* strain (Liao et al., 2015), indicating that the stable resistance to fluoride was the result of genetic mutations.

Interestingly, the stable fluoride-resistant *S. mutans* strains have only been obtained in a laboratory setting so far. There has been no report of a resistant strain in clinical samples (Liao et al., 2017). This is surprising because fluoride-containing products have been widely applied in daily life. One explanation was that the microbes lost their fitness once they became fluoride resistant. It is known that the antimicrobial-resistant microbes could suffer a decrease in biological fitness (Andersson and Hughes, 2010). These fitness costs made antimicrobial-resistant microbes less competitive than susceptible strains when the selective pressure from antimicrobials was removed, which might eventually result in the loss of the antimicrobial resistance (Andersson and Hughes, 2010). In order to understand the effect of fluoride resistance on bacterial fitness, several laboratory-derived fluoride-resistant *S. mutans* strains were compared to their isogenic wild-type strains on growth, acidogenicity, and surface adherence *in vitro* or in animal models (Rosen et al., 1978; Lau and Kral, 1987; Van Loveren et al., 1991a; Liao et al., 2015). However, the results from these studies were inconsistent. For example, the fluoride-resistant *S. mutans* strain C180-2FR, was reported to be less acidogenic than its parent strain in one study (Van Loveren et al., 1991b), but found equally acidogenic to the same parent strain in another study (Liao et al., 2015). Van Loveren et al. (1991a) studied the competition between C180-2FR and its parent strain C180-2 in a rat model. The fluoride-resistant C180-FR strain colonized less and was eventually outgrown by its parent strain. However, Hoelscher and Hudson (1996) characterized another fluoride-resistant *S. mutans* isolate (NCH105) and reported that this strain adhered to the tooth surface to the same extent as the parent strain (UA130). Differences in experimental design or different strains used in these studies may be the reasons for these discrepancies. As a result, it is hard to draw definitive conclusions about the fitness costs of fluoride resistance.

The aim of this study was to investigate the fitness of two fluoride-resistant *S. mutans* strains (UAFR and UF35) in biofilms, using the isogenic wild-type stain UA159 as a reference. The fluoride-resistant strain UAFR was created by culturing UA on agar plates containing increasing concentrations of fluoride (Zhu et al., 2012), whereas strain UF35 was engineered by changing a single nucleotide (-44A→C) in the promoter region *mutp* of UA159 (Liao et al., 2016). Both strains displayed a slower growth rate than the wild-type strain in suspensions. *S. mutans* biofilms rather than planktonic cultures were examined, because biofilms better mimic the bacterial life-style in dental plaque. Moreover, various bacterial growth environments in an oral cavity were simulated by growing bacterial cells under either a constantly neutral pH or pH-cycling conditions. The pH-cycling consists of a period of 8 h at neutral pH and a period of 16 h at pH 5.5.

## Materials and Methods

### Bacterial Strains and Growth Conditions

The strains used in this study were the fluoride-sensitive *S. mutans* strain UA159 and two fluoride-resistant strains (UF35 and UAFR). The construction of strain UF35 was described in Liao et al. (2016). The strain UAFR (accession: NZ_CP007016.1) was kindly provided by Professor Zhimin Zhang (Jilin University, Changchun, China). All strains were routinely maintained on Brain-Heart Infusion (BHI) agar in a jar filled with anaerobic gas (80% N_2_, 10% CO_2_, and 10% H_2_) at 37°C. The medium for biofilm formation was a semi-defined biofilm medium (BM) (Deng et al., 2009). The pH of BM was adjusted either to 7.0 by adding 76 mM K_2_HPO_4_ and 15 mM KH_2_PO_4_, or to 5.5 by adding 100 mM acetic acid. The specific pH used during biofilm formation is explained in detail below. BM with 0.4% glucose (BMG) or 0.2% sucrose (BMS) was used as a medium for the growth of pre-cultures and biofilm formation, respectively.

### Biofilm Formation

**FIGURE 1:**
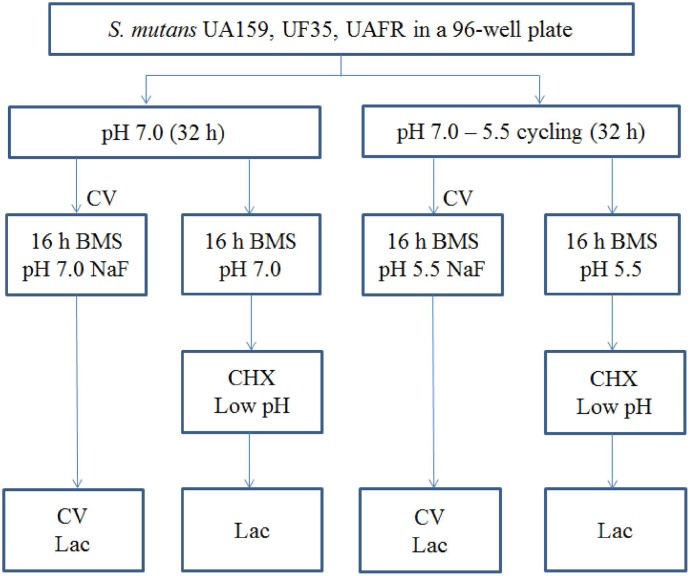
The experimental design of the biofilm formation and treatment. CV, crystal violet assay; Lac, lactic acid quantification.

Biofilms were formed in an active attachment model to avoid bacterial sedimentation (Li et al., 2014). This model consists of a standard 96-well microtiter plate and a lid with 96 polystyrene pegs that fit into the wells (Nunc^TM^, Roskilde, Denmark). Overnight (16 h) cultures of *S. mutans* UA159, UF35, and UAFR in BMG were adjusted to a final OD_600_ of 0.035 in fresh BMS (pH 7.0). The optical density at 600 nm was used to measure bacterial growth of the overnight cultures only. Two hundred microliters of each culture was dispensed into each well of the microtiter plates. The lid with the pegs was then placed over the wells. The plates were incubated anaerobically. After 8 h, half of the biofilms, which formed on the pegs, were transferred to wells containing BMS at pH 7.0, whereas the other half were transferred to BMS at pH 5.5. The biofilms were further incubated for 16 h. Then the biofilms were transferred to wells containing BMS at pH 7.0 for another 8 h. Part of these 32-h biofilms were exposed to NaF. The others were transferred to either BMS at pH 7.0 or BMS at pH 5.5 for an additional 16 h. These 48-h biofilms that were not exposed to NaF were challenged by either chlorhexidine (CHX) or low pH. The schema of biofilm formation and biofilm processing is illustrated in **Figure 1**. All experiments were repeated three times. For each test condition, four replicates were used in every experiment.

### Sodium Fluoride (NaF) Treatment

An initial experiment was carried out in our laboratory to determine the appropriate duration for the NaF treatment. The results indicated that the biofilms displayed detectable phenotypic changes after incubation with NaF for 16 h (data not shown). Therefore, NaF was added to the growth medium at 32 h and its effects on biofilm formation and lactic acid production was subsequently examined after 16 h of incubation. In detail, the 32-h biofilms were incubated in BMS containing various concentrations of NaF (0–12.5 mM) for 16 h. The pH of the BMS was 7.0 for the biofilms formed in constantly neutral pH and 5.5 for the biofilms formed in the pH-cycling condition. The biofilm formation in NaF-medium over 16 h was quantified by a crystal violet assay. The capability of the 48-h biofilms to produce lactic acid was also tested.

### Chlorhexidine (CHX) Treatment

The 48-h biofilms that were not exposed to the NaF (see **Figure 1**) were inserted into wells containing 200 μL CHX solutions at concentrations of 0, 0.01, 0.02, and 0.04% for 5 min. The CHX solutions were prepared from a 20% chlorhexidine digluconate solution (Sigma–Aldrich, St. Louis, MO, United States). The treatment was stopped by inserting the biofilms into a neutralizing solution (6% Tween 80, 0.6% lecithin, and 0.068% potassium phosphate, pH 7.0) for 10 min (Kara et al., 2006). Thereafter, the capability of the biofilms to produce lactic acid was evaluated.

### Low-pH Challenge

The 48-h biofilms that were not exposed to the NaF treatment (see **Figure 1**) were rinsed with sterile distilled water and then inserted into wells containing an acid solution at pH 3.0. The acid solution was prepared from 40 mM potassium phosphate/citrate buffer (Svensater et al., 1997). The duration of the treatment varied depending on the conditions of biofilm formation. The biofilms formed in the constantly neutral pH condition were treated for 1, 3, and 5 min, whereas the biofilms formed in the pH-cycling conditions were treated for 5, 10, and 30 min. The treatment was terminated by inserting the biofilms into a 300 mM HEPES [4-(2-hydroxyethyl) piperazine-1-ethanesulfonic acid] buffer solution (pH 7.0) for 10 min. The lactic acid production of the biofilm was evaluated directly after the treatment.

### Crystal Violet Staining Assay

A crystal violet staining assay (Li et al., 2014) was used to quantify the biomass of the biofilms with or without NaF treatment. The pegs with biofilms were first inserted into a 0.01% crystal violet solution (200 μL/well) for 5 min, washed twice with distilled water, and then inserted into 2% sodium deoxycholate to destain for 5 min. The absorbance of the used destaining solution was measured at 608 nm using a spectrophotometer (Spectramax Plus, Molecular Device, Sunnyvale, CA, United States). The change in biomass during the 16-h overnight incubation was calculated as: the OD_608_ of 48-h biofilms minus the OD_608_ of 32-h biofilms. The data are presented as the percentage of biofilm formation in a NaF group relative to that in the corresponding non-NaF treated control group.

### Lactic Acid Quantification

To quantify the capability of the biofilms to produce lactic acid, the pegs with biofilms were incubated in an assay buffer containing 1% glucose (200 μL/well) for 1 h at 37°C. The assay buffer was prepared from BM without yeast extract to avoid bacterial growth during the 1-h incubation (pH 7.0). The lactic acid concentration in the buffer solution was quantified by an enzymatic-spectrophotometric method (Gutmann and Wahlefeld, 1974). This method is based on the enzymatic conversion of L-lactate to pyruvate with the concomitant reduction of NAD to NADH. The increase in absorbance at 340 nm is proportional to NADH formation. The data is presented as the percentage of lactic acid production of each treated group relative to the corresponding non-treated control groups.

### Viable Cell Counts

Each individual peg with a biofilm on it was cut off from the lid with a sterile scalpel without disturbing the biofilms and placed in 1 mL CPW (5 g yeast extract, 1 g peptone, 8.5 g NaCl, and 0.5 g L-cysteine hydrochloride per liter, adjusted to pH 7.3). Biofilms were dispersed by sonication on ice for 1 min with 1 s pulses at an amplitude of 40 W (Vibra cell^TM^, Sonics and Materials Inc., United States). Ten-fold serially diluted samples were plated onto BHI agar plates (100 μL/sample). The plates were incubated anaerobically for 3 days and colony-forming units (CFUs) were counted.

### Statistical Analysis

The data were analyzed with the Statistical Package for Social Science (SPSS, Version 20.0). One-way analysis of variance (ANOVA) was used to compare the biofilm formation of the three *S. mutans* strains at 32 and 48 h, followed by a Bonferroni’s *post hoc* test. An independent Student’s *t*-test was further applied to compare the 32- and 48-h biomass formations of each strain. The difference was considered significant if *p* < 0.05. The percentage of biomass increase and lactic acid production relative to the control group of these three *S. mutans* biofilms to NaF, CHX, and low-pH treatment were analyzed by a two-way ANOVA analysis, using strains and treatment conditions as the independent variables. When the interaction between strains and treatment conditions was significant, new two-way ANOVA analyses were carried out for each of the three pairs of strains. The difference was considered significant if *p* < 0.016 after Bonferroni correction. All tests were performed for the biofilms formed in the constantly neutral pH and the biofilms formed in the pH-cycling separately.

## Results

In this study, three aspects of fitness were examined for all tested strains in the absence of NaF: biofilm formation, response to CHX treatment, and response to a low-pH challenge. In addition, the inhibitory effect of NaF on biofilm formation and lactic acid production was examined for these three strains to confirm the fluoride resistance of UF35 and UAFR in biofilms.

### Biofilm Formation

**FIGURE 2:**
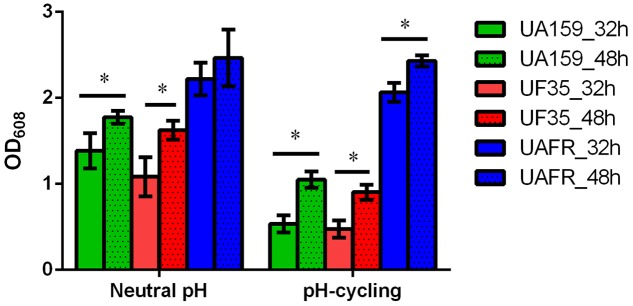
Biomass of 32- and 48-h biofilms. The biofilms of *Streptococcus mutans* UA159, UF35 and UAFR were formed under either a constantly neutral pH or a pH-cycling condition for 32 and 48 h. The biomass was quantified by the crystal violet staining assay and presented as the OD value at 608 nm. ^∗^Indicates the significant difference between the 32- and 48-h biofilms of each strain, *p* < 0.05.

The capability of biofilm formation of three strains, measured with the crystal violet staining assay, is presented in **Figure 2**. The biomass of these strains was measured after 32 and 48 h of incubation. Irrespective of the pH regime during biofilm formation and age, UAFR produced significantly more biomass than UF35 and UA159, whereas the latter two strains did not differ in biofilm formation. The viable-cell plate counts of the 48-h biofilms supported the results from the crystal violet staining assay. The log CFU per biofilms were: 8.1 ± 0.5 (neutral pH), 7.8 ± 0.3 (pH-cycling) for UAFR; 7.1 ± 0.4 (neutral pH), 7.3 ± 0.1 (pH-cycling) for UF35; and 7.4 ± 0.3 (neutral pH), 7.2 ± 0.3 (pH-cycling) for UA159. In accordance with the differences in biomass and CFU counts, the lactic acid production of the 48-h UAFR biofilms was also significantly higher than the other two biofilms. No difference in lactic acid production was observed between UF35 and UA159 biofilms regardless of the growth conditions (data not shown).

### Response of Biofilms to CHX and Low-pH Challenge

**FIGURE 3:**
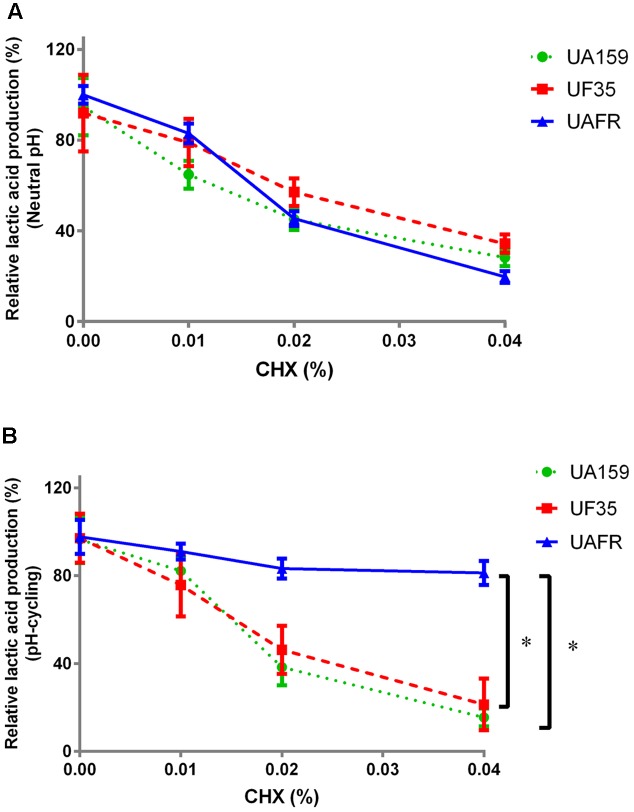
Response of 48-h biofilms to CHX treatment. The 48-h biofilms of *S. mutans* UA159, UF35, and UAFR were treated by CHX for 5 min. **(A)** The biofilms were formed under constantly neutral pH condition. **(B)** The biofilms were formed under pH-cycling condition. The data are presented as the percentage of lactic acid production in the chlorhexidine (CHX) treated groups relative to the corresponding non-treated control group. ^∗^Indicates the significant difference between treatment response of each strain, *p* < 0.016.

**FIGURE 4:**
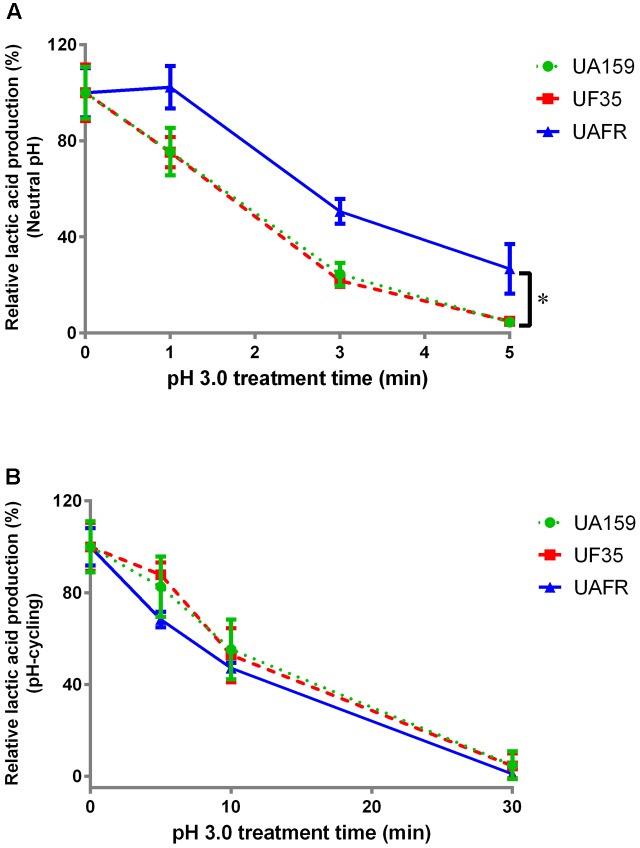
Response of 48-h biofilms to low-pH challenge. The 48-h biofilms of *S. mutans* UA159, UF35, and UAFR strains were treated with pH 3.0 buffer solutions for various periods of time. **(A)** The biofilms were formed under constantly neutral pH condition. **(B)** The biofilms were formed under pH-cycling condition. The data are presented as the percentage of lactic acid production in low-pH treated groups relative to the corresponding non-treated control group. ^∗^Indicates the significant difference between treatment response of each strain, *p* < 0.016.

As stated above, *S. mutans* UAFR biofilms produced significantly more lactic acid than UF35 and UA159 biofilms. Therefore, the treatment efficacies of CHX and low pH are presented as the percentage reduction in lactic acid production of the treated samples relative to the non-treated samples (**Figures 3**, **4**).

**Figure 3** shows that the reduction in lactic acid production in biofilms was enhanced with increasing concentrations of CHX. Under constantly neutral pH, the CHX concentration-dependent reduction in UAFR biofilms seemed to be the strongest among the three strains, but the differences did not reach statistical significance (**Figure 3A**). However, under pH-cycling, this reduction in UAFR biofilms was significantly less than the reduction in the other two biofilms (**Figure 3B**). UF35 biofilms showed similar responses to the CHX treatment as the wild-type UA159 biofilms.

With increasing durations of the low-pH challenge, the reduction in lactic acid production in biofilms became more obvious (**Figure 4**). Again, *S. mutans* UAFR biofilms behaved differently from UF35 and UA159 biofilms, but the difference was only observed for the biofilms formed under constantly neutral pH condition (**Figure 4A**). Under this condition, UAFR biofilms displayed a delayed response (longer than 1 min) to the low-pH challenge, whereas the other two biofilms already showed reductions in lactic acid production after being treated for only 1 min. Moreover, the effect of the duration of the low-pH challenge varied upon the conditions of biofilm formation. When the biofilms were formed in the constantly neutral pH condition, the low-pH challenge reduced the lactic acid production of all biofilms to undetectable levels within 5 min, whereas this took 30 min when the biofilms were formed in pH-cycling condition.

### Response of Biofilms to NaF Treatment

**FIGURE 5:**
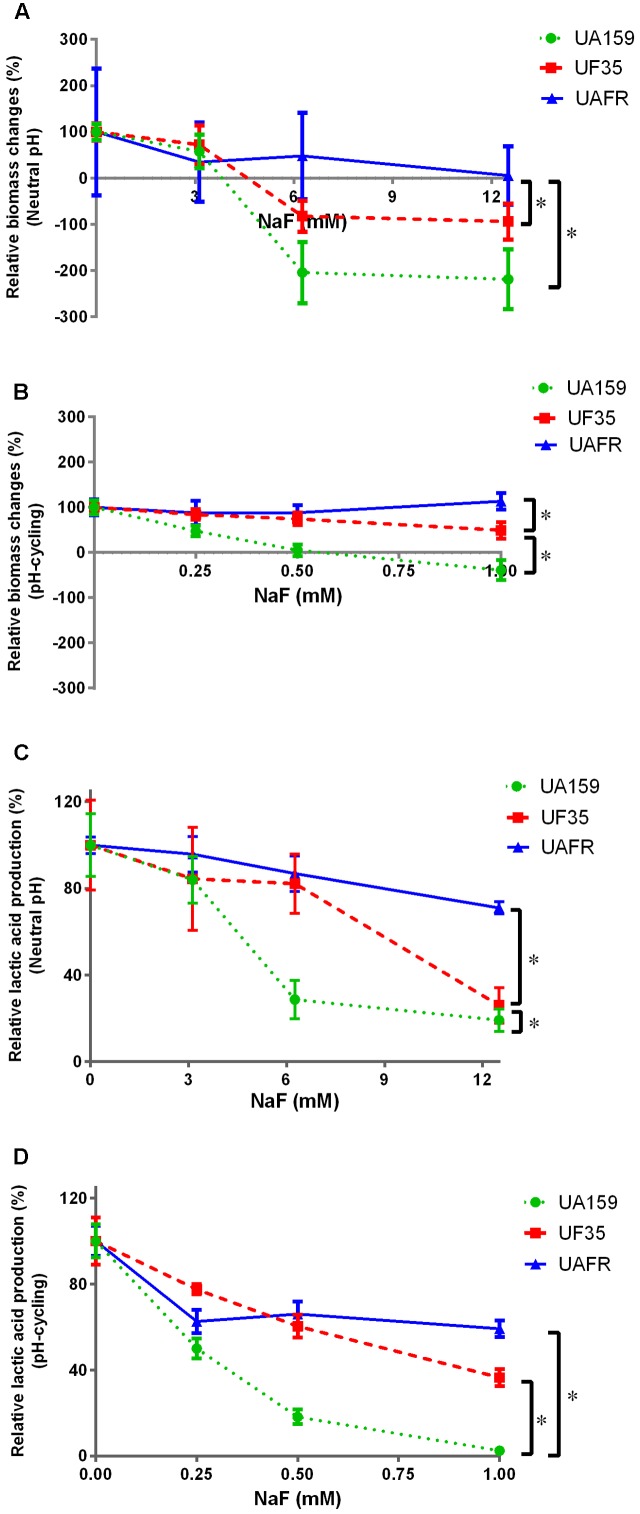
Response of various biofilms to 16-h NaF incubations. The 32-h biofilms of *S. mutans* UA159, UF35, and UAFR were formed with various concentrations of NaF for 16 h under either constantly neutral pH **(A,C)** or pH-cycling condition **(B,D)**. The biomass **(A,B)** and lactic acid production **(C,D)** of the biofilms are shown. The data are presented as the percentage of biofilm formation or lactic acid production in the NaF treated groups relative to the corresponding non-treated control group. ^∗^Indicates the significant difference between treatment response of each strain, *p* < 0.016.

To confirm that the UF35 and UAFR biofilms are indeed less sensitive to fluoride than the wild-type UA159 biofilms, we examined the responses of these three biofilms to a growth medium containing NaF (**Figure 5**). The NaF treatment did not affect the biofilm formation of UAFR, but reduced that of UF35 and, to a larger extent, that of UA159, in a dose-dependent manner (**Figures 5A,B**). In terms of lactic acid production, **Figure 5C** shows that under the neutral pH condition, UAFR biofilms were affected the least, followed by UF35 and then by UA159. The lactic acid production of UA159 biofilms dropped to around 28% of the non-treated group at 6 mM NaF, whereas that of the UF35 biofilms dropped to a level similar to the UA159 biofilms at 12 mM NaF. The lactic acid production of UAFR biofilms did not drop below 70%, even at 12 mM NaF. **Figure 5D** also shows a NaF concentration-dependent reduction in lactic acid production among three biofilms with one exception. At the pH-cycling condition, the reduction of lactic acid production in UAFR biofilms was only observed at 0.25 mM NaF, and no additional reduction could be observed with increasing NaF concentrations. The level of NaF resistance of three strains is generally ranked as follows: UAFR > UF35 > UA159.

Because the treatment efficacy for NaF is known to be pH-dependent, the tested concentration range of NaF differed in the biofilms formed under two pH conditions, 3–12 mM for constantly neutral pH and 0.25–1 mM for the pH-cycling.

## Discussion

In this study, we investigated the fitness of two *S. mutans* fluoride-resistant strains using their fluoride-sensitive wild-type parent strain as a reference. We measured the response of *S. mutans* strains to several concentrations of various antimicrobials. Instead of comparing the responses of *S. mutans* strains at each concentration, we examined the concentration-dependent responses. Our data showed that independent of pH conditions during biofilm formation, neither of the fluoride-resistant strains has a reduced fitness as compared to the fluoride-sensitive strain. To the contrary, one of the strains, UAFR, displayed stronger fitness in biofilm formation, resistance to CHX (pH-cycling only; **Figure 3B**), and resistance to low-pH challenge (neutral pH only; **Figure 4A**) than the wild-type strain and the other fluoride-resistant strain, UF35, in absence of fluoride. Moreover, the UAFR strain coped better with the NaF treatment than UF35. As stated above, these two fluoride-resistant strains differ in the number of chromosomal mutations. Strain UF35 contains one mutation in the promoter region of the fluoride antiporter-coding genes and resists fluoride challenge through up-regulation of fluoride antiporters (Liao et al., 2016). Strain UAFR was obtained by stepwise selection on agar plates containing increasing concentrations of fluoride (Zhu et al., 2012). The genetic mechanism of fluoride resistance in UAFR is still unknown. We have identified up to 21 single nucleotide polymorphisms (SNPs) in the UAFR genome by comparing its genome sequence with that of the wild-type strain UA159. One of these SNPs is located in the same promoter region of the fluoride antiporter genes as in the UF35 strain (data not shown). The differences in fitness and in fluoride resistance between UF35 and UAFR may be related to the number of chromosomal mutations in their genomes. In a previous study, a series of isogenic *Escherichia coli* strains carrying up to five fluoroquinolone resistance mutations were constructed and examined for the fitness cost resulting from fluoroquinolone resistance (Marcusson et al., 2009). The strains containing more than three mutations possessed a better fitness in growth rate in the absence of fluoroquinolone than those containing less than three mutations. In our study, the additional mutations in UAFR may lead to the observed improvement in fitness, including enhanced biofilm formation and resistance to CHX.

The fitness of a bacterium can be influenced by its growth conditions (Bjorkman et al., 2000). In this study, we observed that strain UF35, when grown in biofilms, responded similarly to the low-pH challenge as the wild-type strain, whereas it was previously found to be more susceptible to the same treatment than the wild-type strain when grown in suspension (Liao et al., 2016). Thus, the bacterial lifestyle can lead to differences in fitness. Similarly, we observed that the resistance of UAFR biofilms to CHX and a low-pH challenge also depended on the conditions of biofilm formation. The higher resistance of UAFR biofilms to a 1-min low-pH challenge could be due to the higher biomass of UAFR as compared to the wild-type strain. The treatment time (1 min) may have been too short to allow effective penetration of the acid solution into the dense biofilm layer. However, the resistance of UAFR to CHX treatment when the biofilms were formed in pH-cycling condition cannot be simply explained by its high biomass, because the same concentrations of CHX was applied to the biofilms formed in either pH condition. Further studies are necessary to investigate the role of the different gene mutations in the UAFR strain’s resistance to CHX treatment.

As shown in the treatment design of this study (**Figure 1**), the duration of the low-pH challenge was much longer for the biofilms formed under pH-cycling conditions up to 30 min than for the biofilms formed under constantly neutral pH conditions up to 5 min. This increased resistance to the low-pH challenge might be related to the acid tolerance level of the biofilm formed in pH cycling conditions. It is known that *S. mutans* is able to alter its physiology under acidic conditions in order to survive: this adaptive response is referred as the acid tolerance response (ATR) (Welin-Neilands and Svensater, 2007). Several studies have demonstrated that planktonic cells and cells in biofilms survived acid killing better after a prior exposure to low but non-lethal pH values (McNeill and Hamilton, 2003; Welin-Neilands and Svensater, 2007). In our experiments, the biofilms formed in pH-cycling conditions have been exposed to pH 5.5 for 16 h before the acid killing treatment, which might trigger the ATR and result in stronger resistance to acid killing.

This study showed that a duration of 16 h was required to observe an inhibitory effect of NaF on the biofilms. It also determined the inhibitory concentrations of NaF on biofilms, being 3–12 mM when the pH of NaF solutions was neutral and 0.25–1 mM when the pH of NaF solutions was 5.5. Although these are *in vitro* findings, they are helpful in estimating the selective pressure that a daily usage of NaF can impose on the oral microbes. *In vivo*, the concentration of fluoride remaining in dental biofilms after application of fluoride-containing products was reported to be between 0.06 and 0.3 mM F (Duckworth et al., 1987; Naumova et al., 2012). In a healthy oral ecosystem, where the pH of dental biofilms is mostly neutral, the daily application of fluoride would not impose any selective pressure, because our data showed the effective treatment concentrations of NaF should be above 3 mM (57 ppm F) when the pH of NaF solution was neutral. However, this might not be the case for the dental biofilms in caries-susceptible subjects or at approximal sites, where the pH of these biofilms often remains low for an extended period of time (Jensen and Schachtele, 1983). Our data showed that the fluoride-resistant biofilms (UF35) produced significantly more lactic acid (1.05 ± 0.07 mM) than the fluoride-sensitive UA159 biofilms (0.76 ± 0.01 mM) in pH cycling conditions after being treated with 0.25 mM NaF (4.75 ppm F). In the caries-susceptible biofilms *in vivo*, the daily application of fluoride would likely promote fluoride-resistant strains. Therefore, we could not exclude the possibility that the lack of clinical fluoride-resistant *S. mutans* isolates might be related to low selective pressure despite the daily fluoride usage. Fluoride-resistant strains might only be present in a specific niche in the oral cavity.

In summary, this study compared the fitness of two fluoride-resistant strains with their wild-type fluoride-sensitive parent strain. In the absence of fluoride, neither fluoride-resistant strain showed compromised fitness as compared to the wild-type strain. In addition, the fluoride-resistant strain that contains multiple chromosomal mutations exhibited better fitness than the wild-type strain and the other fluoride-resistant strain that contained only a single chromosomal mutation. To understand better the fate of fluoride-resistant strains in an oral cavity, the fitness of these fluoride-resistant strains may be examined in a model that resembles the oral environment. For example, in a multi-species biofilm model, the fitness can be studied in the presence of host factors and other oral microorganisms. In addition, the competition between fluoride-resistant and fluoride-sensitive strains could be examined in such a model.

## Author Contributions

Conceived and designed the experiments: YC, XW, and DMD. Performed the experiments: YC, HL, and DMD. Analyzed the data: YC, YL, BB, and DMD. Drafted the manuscript: YC, YL, BB, and DMD. Critically revised the manuscript: XW, WC, and CVL. All the authors approved the final version of the manuscript and agreed to be accountable for all aspects of the work.

## Conflict of Interest Statement

The authors declare that the research was conducted in the absence of any commercial or financial relationships that could be construed as a potential conflict of interest.
